# Detection of an invasive aquatic plant in natural water bodies using environmental DNA

**DOI:** 10.1371/journal.pone.0219700

**Published:** 2019-07-12

**Authors:** Marc B. Anglès d’Auriac, David A. Strand, Marit Mjelde, Benoit O. L. Demars, Jens Thaulow

**Affiliations:** 1 Norwegian Institute for Water Research (NIVA), Oslo, Norway; 2 Norwegian Veterinary Institute, Oslo, Norway; University of Hyogo, JAPAN

## Abstract

The ability to detect founding populations of invasive species or rare species with low number of individuals is important for aquatic ecosystem management. Traditional approaches use historical data, knowledge of the species’ ecology and time-consuming surveys. Within the past decade, environmental DNA (eDNA) has emerged as a powerful additional tracking tool. While much work has been done with animals, comparatively very little has been done with aquatic plants. Here we investigated the transportation and seasonal changes in eDNA concentrations for an invasive aquatic species, *Elodea canadensis*, in Norway. A specific probe assay was developed using chloroplast DNA to study the fate of the targeted eDNA through space and time. The spatial study used a known source of *Elodea canadensis* within Lake Nordbytjern 400 m away from the lake outlet flowing into the stream Tveia. The rate of disappearance of *E*. *canadensis* eDNA was an order of magnitude loss over about 230 m in the lake and 1550 m in the stream. The time series study was performed monthly from May to October in lake Steinsfjorden harbouring *E*. *canadensis*, showing that eDNA concentrations varied by up to three orders of magnitude, peaking during fall. In both studies, the presence of suspended clay or turbidity for some samples did not hamper eDNA analysis. This study shows how efficient eDNA tools may be for tracking aquatic plants in the environment and provides key spatial and temporal information on the fate of eDNA.

## Introduction

The detection of invasive species is often challenging during the initial settlement phase due to low numbers of founding individuals [1, 2]. Early warning detection is key for managers to respond with best possible measures to prevent potential negative outcome to endemic fauna and biota [3, 4]. Traditional field surveys with visual inspection require taxonomic knowledge of the invading species and biotope knowledge combined with risk of dispersal to assess where to search [5]. Environmental DNA (eDNA), which is shed by all living organisms in the environment, is increasingly used for the detection of elusive or low abundant species and has been shown to be equally or more sensitive than traditional surveying methods [6, 7]. So far, the majority of developed eDNA single species detection methods have primarily focused on aquatic animals including mammalians [8, 9], fish [8, 10–17] Molluscs [18, 19], crustacea [20–22], amphibians [23, 24] reptiles [25–27] and insects [28–30]. Detection of aquatic plant eDNA has been scarce in comparison [31]. In the wild it was first demonstrated for *Egeria densa* and *Hydrilla verticillata* in small Japanese ponds (83–6000 m^2^) where the presence of the species was visually confirmed and compared to past distribution records [32, 33]. Another study successfully tested eDNA detection of *H*. *verticillata* in north American rivers and lakes [34] and three studies detected eDNA where the species had not yet been observed [31, 34, 35]. Several laboratory mesocosm experiments have tested the changes in aquatic plant eDNA over time, during and after introduction, and with and without grazers [32–34]. However, to the best of our knowledge, no temporal or spatial studies have yet been conducted in the wild for studying potential seasonal variations and transportation of aquatic plants eDNA.

The Canadian pondweed *Elodea canadensis* Michaux, originates from North America and has colonized Europe at least since it was first recorded in Ireland in 1836 and Britain in 1842 [36]. The species was first observed in Norway in 1925, and has now spread to more than 100 southern Norwegian water bodies [37, 38] and has become the most widespread aquatic invasive macrophyte in Europe [39]. This is a rooted submerged flowering plant growing mostly in standing waters (canal, ditches, ponds, lakes). The species can produce 2–3 m long shoots and in clear water can grow down to 5–6 m depth [40]. The growing season starts in April–May and normally last until September-October, with biomass peak in July–August. However, in some lakes, e.g. Lake Steinsfjorden, the *Elodea*-stand can survive under ice-cover and collapse the following spring, and new growth develops from the decaying biomass [41, 42]. This species is dioecious, i.e. individual plants have only male or only female flowers, and in Europe male flowers are rarely seen suggesting the plant reproduction is mostly vegetative with overwintering buds and stem fragments [42]. In general *E*. *canadensis* shoots are sensitive to desiccation although apices and vegetative propagule may be more tolerant [43, 44]. These propagules can spread rapidly within lakes and downstream watercourses. Other vectors of dispersion can be by birds [45], but also most likely people through recreational boating, fish farming or angling [37, 46, 47].

This invasive aquatic plant is important for environmental management as it may affect the biodiversity and functioning of freshwater ecosystems where it grows in high abundance [40, 41, 48, 49].

In this study we developed molecular markers for *E*. *canadensis*, which we used for eDNA detection in two distinct studies and sites. The first study (site 1) is a spatial transect throughout an entire river catchment where eDNA results are compared with visual survey results and historical data to assess the spreading of the species. The aim of this first study is to assess how eDNA corroborates with visual results and to construct a gross estimate of eDNA disappearance during transportation. The second study (site 2) analyses eDNA signal strength through seasons at a location in a lake invaded by *E*. *canadensis* (time series) to assess how seasonality may affect eDNA signal strength of a sessile aquatic plant target.

We discuss how our findings may be useful for designing and interpreting aquatic plants eDNA surveys for management purposes.

## Materials and methods

### eDNA practices

Good practices for eDNA work [50] were implemented both in the field, for sample collection, filtration, and transport, as well as in the laboratory for DNA extraction and qPCR analysis. For optimum DNA preservation water samples were either transported on ice and processed with 24 h (site 1) or filtered on site (site 2). Virkon S (LANXESS Deutschland GmbH, Cologne, Germany) and 10% bleach solutions were used for neutralising DNA on surfaces including sample containers, field material and work benches in the laboratory. Prior knowledge of observed presence of the target species was used for ordering sampling in the field at site 1, collecting last known positive sites in the following way: # 8, 5, 4, 2, 1, 3, 7, 9, 6 & 10 (see Table 1.) Negative controls, minimum 2 and up to 6 per qPCR plate, were included in all runs. Due to the ecology of the targeted species, a sessile aquatic plant, allochthonous DNA is not foreseen to occur or interfere at the 2 studied sites. Calibration curves using standardized *E*. *canadensis* genomic DNA were used for quantification in fg/mL (S1 Fig). Quantification in target copy number using target qPCR products for the calibration curves was not used in order to reduce cross contamination risks. Probe-based qPCR technology was selected as recommended when using eDNA for single species detection [50]. Specificity of the *E*. *canadensis* probe assay was challenged both *in-silico* and *in-vitro* (see below for details). However, testing for possible inhibition was not performed albeit results indicate that no or little inhibition was present.

**Table 1:** Sampling information for the eDNA spatial transect (sampled 20.09.2018).

ID	Site name	Previous known presence	GPS	Water body type
#1	Nordbytjernet	Present	60.1479309, 11.1538417	Lake
#2	Kværndalsbekken	Unseen	60.1555710, 11.1578630	Stream, 50 m downstream from lake outlet
#3	Tveia (Gropavegen)	Unseen	60.1477098, 11.1383513	Stream, 1400 m downstream from lake outlet
#4	Tveia (Nordre Haga)	Unseen	60.1329602, 11.0802854	Stream, tributary of Leira
#5	Leira (Tangen)	Unseen	60.1715632, 11.0296680	River
#6	Leira (Tuen)	Unseen	60.0766315, 11.0621212	River
#7	Leira (Kråkfoss)	Unseen	60.1217685, 11.1129639	River
#8	Gjermåa (Tangen)	Unseen	60.2535285, 11.0047371	Stream, tributary of Leira
#9	Merkja	Present	60.0779336, 11.1031764	River delta
#10	Nitelva (Lillestrøm)	Present	59.9498737, 11.0464909	River

### Study 1: eDNA spatial transect field survey

#### River Leira catchment description

The river Leira is unregulated and drains a catchment area of 663 km^2^. The upper part of the catchment is covered by coniferous forest growing on rocks and moraine deposits. It is characterized by the presence of numerous large lakes, fast flowing and clear waters. The lower part of the catchment is dominated by agriculture, where the solid geology is covered by marine deposits including thick layers of clays. The density of the drainage network is much higher, the water is turbid with very high suspended sediment concentrations during high flow events [51]. The lower part of the Leira is meandering with many oxbow lakes and backwaters and discharges into the River Nitelva, just north of Lake Øyeren receiving the largest European freshwater delta from the River Glomma. Lake Øyeren is protected by the Ramsar convention and with over 40 species it hosts the highest species diversity of aquatic plants in Norway [52].

**Fig 1:**
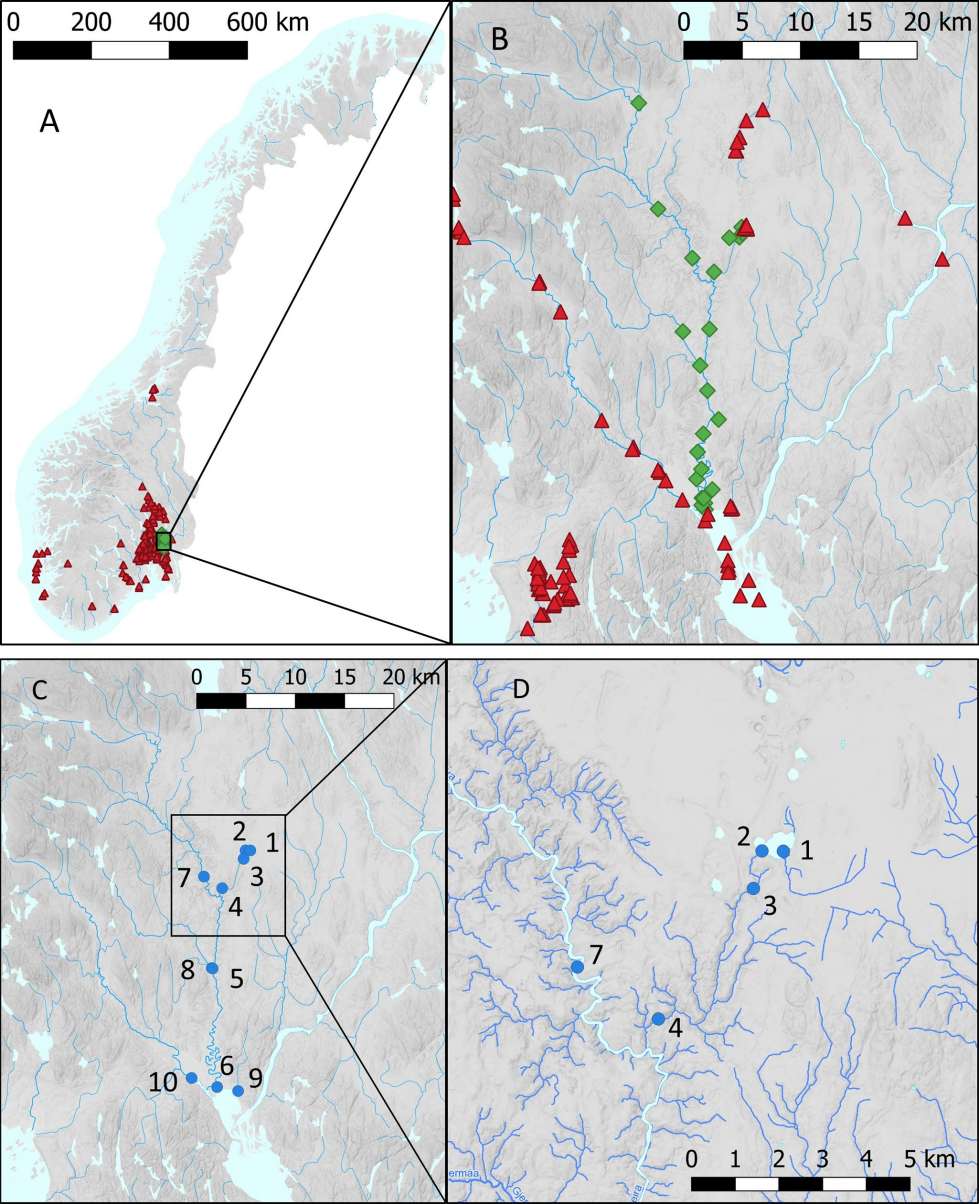
Known distribution of *Elodea canadensis* in Norway and survey locations. In **A**–**B** The red triangular symbols represent localities with known presence of *Elodea canadensis* downloaded from Artsdatabanken (https://artsdatabanken.no/), and the green diamond symbols indicate the sites for the visual survey. In **C**–**D** the blue circle symbols show water sample locations for eDNA analysis (with ID numbers #, see Table 1).

There were two known populations of *E*. *canadensis* in the catchment: Lake Nordbytjern (since 1989) half way up the catchment in the headwaters of a tributary (River Tveia); and a backwater Isakbekken (since 1982) in the lower part of the catchment where the river meanders [53] (Fig 1). Lake Nordbytjern is a small (surface area 0.2 km^2^), high alkalinity and mesotrophic lake. *Elodea canadensis* was also known to be present in the delta area (Nitelva, Svellet, Merkja) and adjacent catchments (Nitelva, Hersjøen and Risa) [53] (Fig 1).

#### Spatial transect field survey description

The design of this study took advantage of prior knowledge of presence of *E*. *canadensis* in the upper and lower parts of the catchment area. The important variations of turbidity across the studied area enabled qualitative assessment of its possible effect on eDNA detection. The clay rivers were generally not a suitable habitat for *E*. *canadensis*. We checked for presence of *E*. *canadensis* in the river at locations spaced every 5–7 km, which is a shorter distance than the spatial autocorrelation of aquatic plant composition in lowland rivers, i.e. 10 km [54]. The lower meandering part of the river Leira has several oxbow lakes, open backwaters and ditches. These stagnant or slow flowing water bodies are generally excellent habitat for aquatic plants and the most at risk of *E*. *canadensis* colonisation due to the proximity of the delta where *E*. *canadensis* is present (e.g. Møller and Rørdam [55]). This is also where the knock-on effect on biodiversity potentially is the highest.

We surveyed 23 sites by wading and/or snorkelling for 15–30 min (see S1 Table) selected for the likelihood of finding new populations of *E*. *canadensis* during summer 2018 (Fig 1). Water samples for eDNA analysis were collected in two 1 L prewashed plastic bottles with a wide neck and transported back to the lab in coolers with ice. Disinfection with Virkon S was carried out in between each sampling station. We took special care that no plant fragments adhered to our equipment.

We collected water samples from lakes and rivers of the Leira catchment area, an adjacent river (Nitelva, ID 10) and the delta area (Merkja, ID 8)–see Table 1 and Fig 1. Water samples were all collected on the 20^th^ September 2018 under stable low flow conditions following a rainfall event. All the samples taken upstream of the marine clay deposits had clear water, but the samples from the lower part of the catchment were very turbid, mostly from suspended clay. The samples were collected just below the water surface (5–20 cm depth). Nitrile gloves were worn for collection of each sample and changed between sample sites. In the laboratory, bottles were wiped with 10% bleach before opening and filtered through a sterile 0.22 μm polyethersulfone disposable Sterivex GP filter with Luer-Lock (Millipore) with sterile disposable 60 mL Luer-Lock Tip syringes (Becton Dickinson). Briefly, the disposable 60 mL syringe is filled with the sample and fitted to the inlet female Luer-Lock of the Sterivex cartridge filter, to push through the water sample. The operation is repeated until the desired volume is filtered or when the capacity of the filter is reached, typically about 1 L for non-turbid samples. The remaining water in the cartridge is removed by using air in the syringe. DNA was further extracted from the Sterivex filters according to Spens, Evans [56].

### Study 2: Lake Steinsfjorden eDNA time series field study

Lake Steinsfjorden is situated in the south-eastern lowland area of Norway, with surface area of 13.9 km^2^ and mean depth of 9.9 m. It is a moderately alkaline and slightly mesotrophic lake. The main watercourse upstream of Lake Steinsfjorden has been heavily infested with *E*. *canadensis* since the early 1960s, and *E*. *canadensis* was first observed in Steinsfjorden in 1978, in the southern and western parts of the lake [48]. From these localities, *E*. *canadensis* spread rapidly within the lake, until the distribution peaked in 1982 [57]. The distribution has remained relatively stable after that [58]. Water samples were collected once a month (May to October) at the surface (~1 m depth) and just above the bottom (~7 cm above, at ~3 m depth), at the same site (60.08175° N 10.337306° E (WGS84)). Due to issues with the pump only bottom samples were collected in June and July. This resulted in a total of ten samples covering the growth and initial decomposition period in Southern Norway with 6 sampling dates over a 5-month period. The eDNA water samples (5 L) were collected by pumping water directly from the lake onto glass-fibre filters (47 mm, 2 μm pore size, AP2504700 Millipore, Billerica, Massachusetts, USA) using a peristaltic pump (Masterflex E/S, Cole-Parmer, Vermon Hills, Illinois, USA) with Tygon tubing (Cole-Parmer) and an in-line filter holder (47 mm, Millipore). Ambient water was pumped through the tubing and filter holder to rinse the system between the bottom and surface samples. After field sampling, a 10% bleach solution was pumped through the tubing and filter holder and the tubing and filter holder was soaked in the bleach solution for 15 minutes to disinfect and remove eDNA traces. A 5% sodium thiosulfate solution was then used to rinse away the bleach. Due to high turbidity only 0,75 L sample was filtered above the bottom in October. Each filter was transferred to a 15 mL sterile falcon tube, stored on ice in a cooling box until transported to the laboratory within 12 hours, and frozen at -20°C. DNA was extracted from the glass-fibre filters using a CTAB extraction protocol, as described in [20]. These samples were initially collected as part of an eDNA study on freshwater crayfish that also inhabit the lake (unpublished) and thus the reason for a different sampling approach than in study 1. One of the benefits of eDNA sampling is the possibility to use the same sample to investigate the presence of several different organism.

### Primer probe assay design and specificity

Alignments, primer and probe design based on the chloroplast intergenic spacer between the *trn*L and *trn*F genes, were made using Geneious R 10.1.3 (https://www.geneious.com) and GeneDoc v2.7 softwares (see Table 2).

**Table 2:** Primers and probe.

Target	Oligonucleotide name	Sequence (5’-3’)	[nM]	Product (bp)
*Elodea canadensis trn*L–*trn*F intergenic spacer	Ec*trn*L_F	TTTCTCCTTCATTGTATTCTTTCACA	500	103
	Ec*trn*L_R	TGTTGATTTCTATCTGTATTGTAGAC	500	
	Ec*trn*L_P	FAM-TCCGAACAGAAATGCCTCTCTCTTATCC	200	

**Fig 2:**
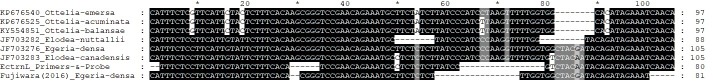
BLAST alignments. Alignment of relevant Hydrocharitaceae sequences obtained by performing a BLAST search using the *Elodea canadensis* product sequence amplified from a locus on the intergenic spacer between *trn*L and *trn*F. Species specific oligonucleotides for *E*. *canadensis* and *Egeria densa* (Planch.) [32] are shown at the bottom.

Specificity of the assay was challenged by testing possible cross amplification with the following macrophytes, many of which are commonly found in Norway: *Potamogeton berchtoldii*, *P*. *obtusifolius*, *P*. *perfoliatus*, *P*. *friesii*, *P*. *pusillus* and *P*. *gramineus*. All six tested macrophytes other than *E*. *canadensis* produced negative results. Although not tested, the assay should also be specific against *Elodea nuttallii* as the 3’ last eight nucleotides of the reverse primer Ec*trn*L_R overlap a gap in the *E*. *nuttallii* sequence. Similarly, the probe Ec*trn*L_P overlaps another eight-nucleotide gap in the *E*. *nuttallii* sequence (Fig 2), further increasing the specificity of the assay.

This was completed with *in-silico* testing using the *E*. *canadensis* qPCR amplicon in a BLAST search [59]. The resulting species matches, all from the Hydrocharitaceae family, were aligned and are shown in Fig 2, confirming the specificity of the chosen oligonucleotides (see discussion). The species *Hydrilla verticillata* also had a partial match, although more divergent, and is included in S1 File along with GenBank access numbers and fasta files of the aligned products. The same sequences were used for designing the specific *E*. *canadensis* probe assay.

### qPCR

A CFX96 thermocycler (Bio-Rad, Hercules, CA, USA) was used to carry out qPCR amplifications with a final reaction volume of 25 μL containing 12.5 μL TaqMan Environmental Mix (ThermoFisher Scientific, Waltham, MA, US), 5 μL sample, 0.165 μL of forward primer (20 μM), 0.165 μL of reverse primer (20 μM), 1 μL probe (5 μM) (LGC Biosearch, Risskov, Denmark) and 5.25 μL sterile deionised water. Final concentrations are indicated in Table 2. A two-step cycling protocol was carried out with a 10 min denaturing step at 95°C, followed by 45 cycles of 95°C for 15 s and 58°C for 60 s. Reference DNA for *E*. *canadensis* was prepared as described previously [60]. Briefly, *E*. *canadensis* material was incubated for 5 minutes at 100°C with 600 μL sodium phosphate buffer (pH 8) in 1.5 ml Eppendorf tubes, and then transferred to a 2 ml cryopreservation tube with 0.5 g zirconium beads and 100 μL 25% sodium dodecyl sulphate added. Bead beating was performed in a Precellys 24 bead beater (Bertin, Technologies, Saint-Quentin, France) as following: 3 x 15 s at 6000 rpm, and 30 s at 6,00 rpm. The samples were then centrifuged (6 min, G = 13700) and DNA was further purified according to [61].

A 8.6 ng/μL stock solution was used to prepare a ten-fold serial dilution for qPCR calibration and calculation of amount of eDNA from each sampling location. The qPCR calibration curves had a 5-log base 10 linear range, a linear regression correlation coefficients (r^2^) greater than 0.99 and an efficiency equal or better than 97.0% (S1 Fig).

In addition to the positive control, negative extraction and blank qPCR controls were also added to each qPCR analysis. All qPCR samples were run in duplicate for Lake Steinsfjorden time series samples and in triplicate for the spatial transect samples.

## Results

### River Leira eDNA spatial transect field survey

**Fig 3:**
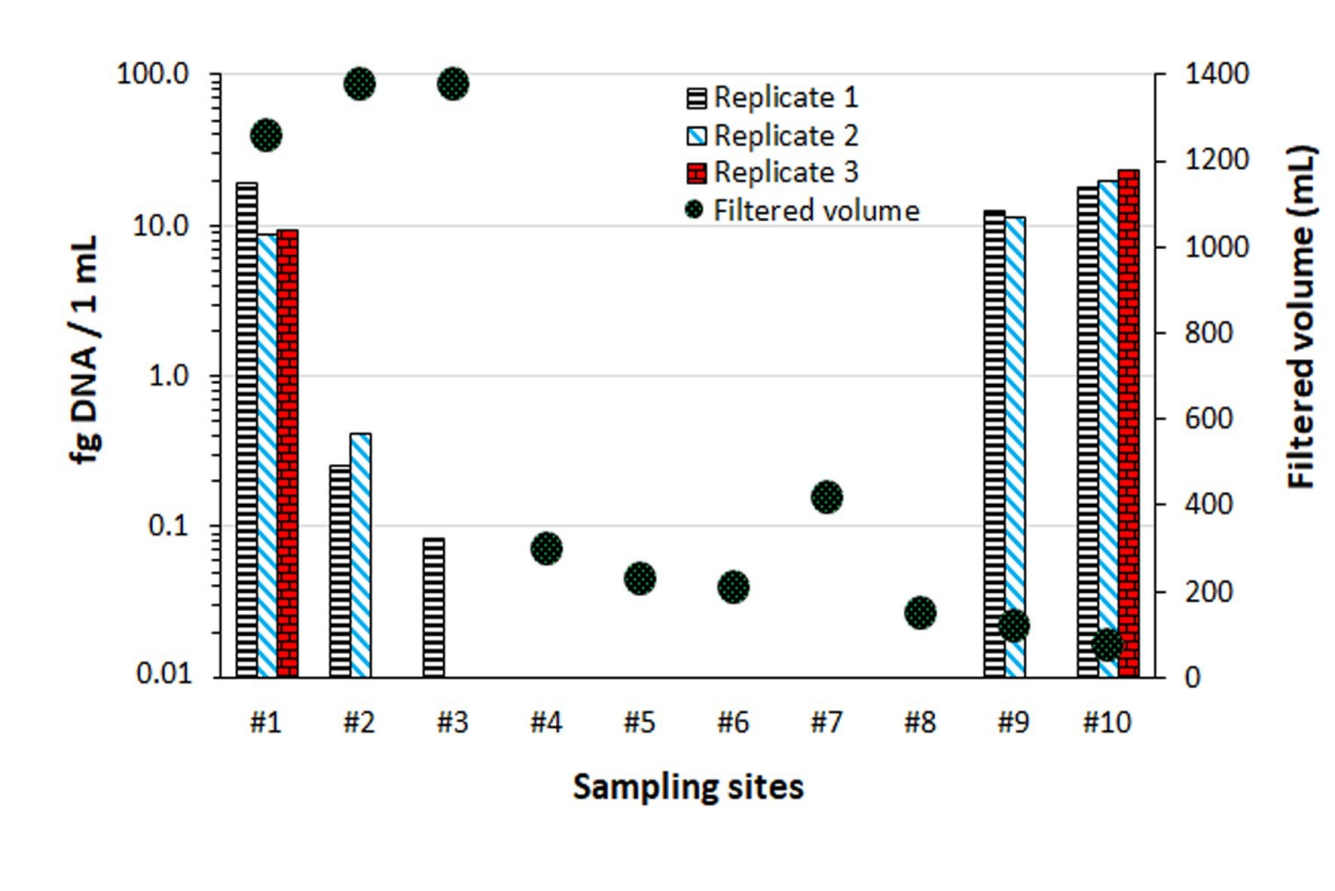
eDNA spatial transect over the Leira catchment. Numeration corresponds to Fig 1 and Table 1. Technical qPCR replicates are shown for each sampling site.

**Fig 4:**
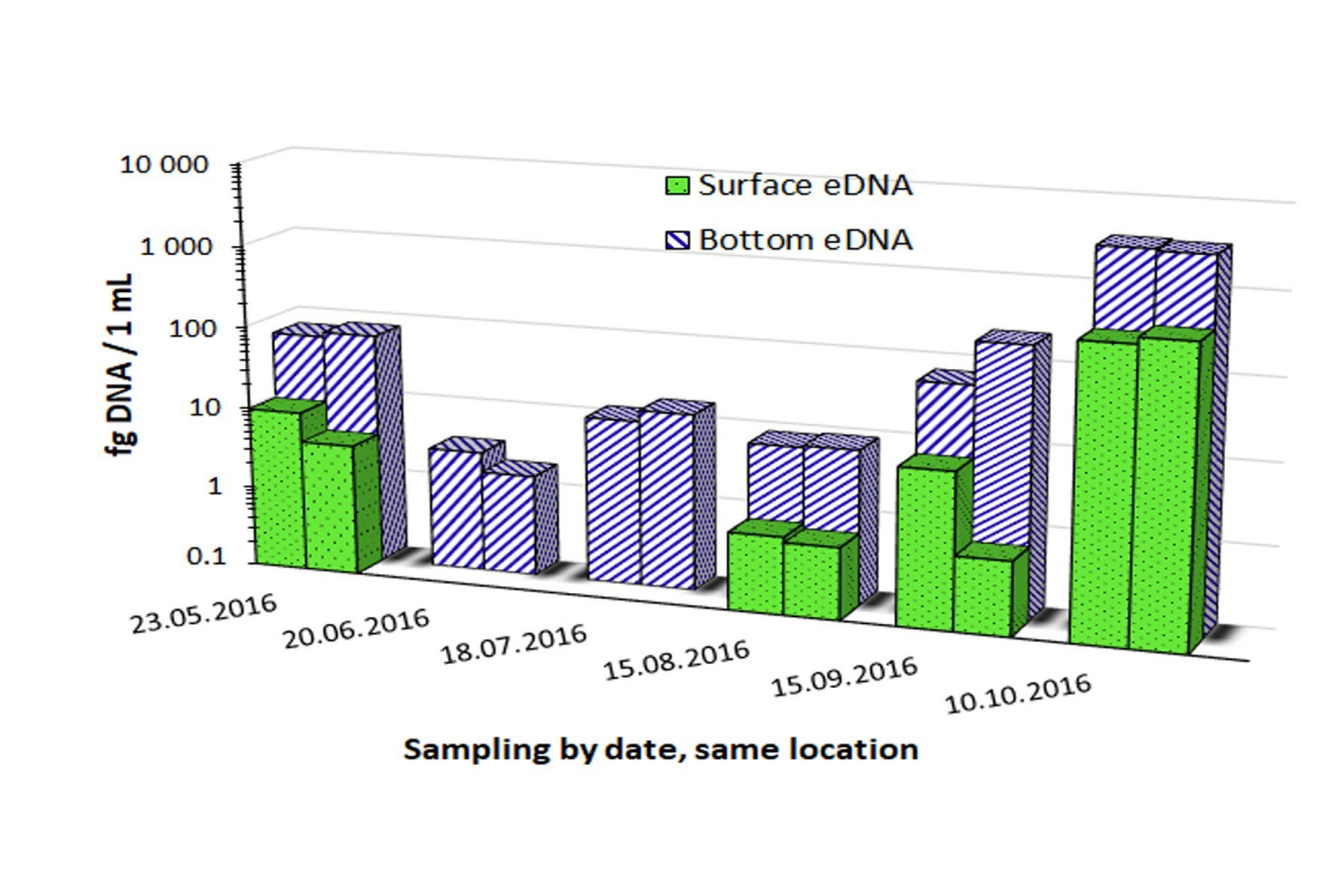
*Elodea canadensis* eDNA time series at Lake Steinsfjorden (one location). No surface eDNA samples were collected on the 20 June and 18 July 2016. Filtered volume for all samples was 5000 mL apart for the 10^th^ October 2016 bottom sample that used only 750 mL due to high turbidity. Technical qPCR replicates (parallels) are shown for each sampling date and depth.

Presence of suspended clay in the lower part of the catchment had a marked incidence on the volumes that could be filtrated from about 1300 mL for the upstream samples down to 75 mL for the last downstream sample (see Fig 3). *Elodea canadensis* eDNA was detected in the sample from the source lake, with known presence of the aquatic plant, yielding almost 10 fg/mL. The detected eDNA quantities decreased in the next two consecutive sampling taken at the outlet of the lake and 1.4 km downstream, to thereafter show negative results for the next five downstream sampling locations (site 2–6, see Figs 1 & 4). The limit of detection was close to 0.1 fg/mL. The last two samples taken close to the mouth of River Leira (Nitelva and Merkja) showed eDNA concentrations exceeding 10 fg/mL despite turbid waters and low filtrated volumes.

### Lake Steinsfjorden eDNA time series field study

All samples were collected at the same location (S2 Fig). All tested samples were positive for eDNA while all negative controls showed no amplification. Large seasonal variations were registered from around 1 fg/mL to over 1000 fg/mL eDNA quantities as shown in Fig 4. Bottom samples yielded more DNA than surface samples for the same date and location. The only turbid sample (October) that could not be filtered more than 750 mL, instead of 5000 mL for all others, also yielded the highest eDNA quantity. Two seasonal peaks were observed, one for the first sampling date in May and the highest for the last sampling date in October.

## Discussion

So far, most eDNA studies have focused on monitoring animal species, and only recently has this powerful method been applied to tracking aquatic plants. Typically, the eDNA is further analysed either by using species specific qPCR or traditional barcoding applied to water [31] for tracking invading or threatened species, or using metabarcoding for water [62] or soil samples [63] for global ecosystem biodiversity monitoring. In this study we have developed a specific probe assay for eDNA detection of the aquatic plant *E*. *canadensis* and used it to conduct a spatial transect field study as well as a time series field study. Variations in turbidity of the water samples along the spatial transect enabled evaluation of its possible interaction on eDNA detection.

### *E*. *canadensis* specific probe assay

In an effort to stimulate the use of eDNA for the detection of invasive aquatic plants Scriver, Marinich [31] used three chloroplast markers for developing species specific assays and concluded that *mat*K was the most likely to provide species specific nucleotides. However, no *Elodea* species were included for which Gantz, Renshaw [34] later developed assays detecting *E*. *canadensis*, *E*. *nuttallii* as well as *Hydrilla verticillata*. They also used the *mat*K marker although the resulting assay that was developed could not differentiate between *E*. *canadensis* and *E*. *nuttallii*. However, a specific assay was reported using the genomic ITS1 marker. Finally, Fujiwara, Matsuhashi [33] used the intergenic spacer between the *trn*L and *trn*F genes for designing an assay for the specific detection of *Egeria densa* [33]. In the present study we also used the *trn*L–*trn*F intergenic spacer to design a probe assay specific for *E*. *canadensis* taking advantage of the two gaps present in the *E*. *nuttallii* sequences when aligned with the *E*. *canadensis* sequences. Although partly overlapping the Fujiwara, Matsuhashi [33] primers and probe, our assay should not cross amplify with *E*. *densa* because two SNPs are present in the 3’ end section of the reverse primer and one SNP is present in the probe (Fig 2). However, this was not tested. BLAST results showed three additional species belonging to the gender *Ottelia* which presented a sequence gap similar to *E*. *nuttallii*, precluding the possibility of annealing with the reverse primer of the *E*. *canadensis* assay (Fig 2). The higher copy number of chloroplast markers *versus* genomic markers usually make them better choices for assays requiring best possible sensitivity, as long as enough variation is present to enable the required specificity. We therefore believe our probe assay is well suited for the specific detection of *E*. *canadensis* from eDNA samples. An overview of species from the Hydrocharitaceae family with existing assays and range of distribution is given in S2 Table.

### eDNA spatial transect study

Due to relatively fast degradation of DNA once it enters the environment [64] species detection can be considered recent as long as sediments have not been disturbed [65]. Due to this breakdown as well as dilution and adsorption, maximum range detection distance away from the target species, especially in running water, is not straight forward [66, 67]. Hence, biomass quantification estimates from eDNA should also take these aspects into consideration [10, 15]. The spatial transect of this study gives a first rough empirical estimate of *E*. *canadensis* eDNA persistence in a stream from a known source lake. The eDNA concentrations decreased (as expected) downstream from the lake with known presence of *E*. *canadensis*. The source population of *E*. *canadensis* in Lake Nordbytjern was situated in the south east corner of the lake, about 400±50 m from the outlet. The extent of *Elodea* population was about 100–500 m^2^ and not very dense (2017 boat survey with a bathyscope). At the time of survey *E*. *canadensis* presented no sign of decay, most of its leaves rather covered by calcium precipitate. Since eDNA was detected at the outlet and 1400 m downstream of the outlet, we can estimate the rate of disappearance of *E*. *canadensis* eDNA per unit distance in the lake and in the stream (S2 File). The rate of disappearance of *E*. *canadensis* eDNA was an order of magnitude loss over about 230 m in the lake and 1550 m in the stream. On average a detectable fragment of *E*. *canadensis* eDNA will travel 650 m in the stream (S2 File). These rates must be seen as first estimations to guide more detailed studies as for other organisms. In two other studies, eDNA from freshwater mussel beds (sessile organism) were detected 1000m downstream in a mesocosm [18] and 1700m downstream of a natural large aggregation [68], which is in the same range as the 1400m found in this study.

### Water turbidity interaction with eDNA detection

Downstream samples of the spatial transect showed large differences in filtration volumes due to the presence of suspended clay in the lower parts of the river system. However, lower filtrated volumes due to turbidity may not necessarily hamper eDNA detection. Indeed, DNA bound on clay minerals (and other particles) can be more resistant to degradation [69] and this could explain the high eDNA concentrations obtained with the very small sample filtered volume performed at the mouth of the studied river. The high concentrations could also be partly explained by the known high densities of *E*. *canadensis* in the River Nitelva and the delta area (Merkja). The lake and stream outlet were situated above the marine clay deposits and had clear water, explaining the high volumes of water filtered at those sites (Fig 3). It would be interesting to measure the level of protection clay may provide to eDNA by assessing and comparing eDNA persistence from a source in either clear or clay-rich water systems. However, not all turbid waters will favour eDNA studies as they may also hinder proper eDNA evaluation of the studied water body [70].

### eDNA time series field study

Variation through time of eDNA for a given species at a defined location is known to be dependent on various factors, in particular seasonal activity [71] as well as migration patterns for animals. Aquatic plants, with maybe the exception of floating plants, are less prone to geographic displacements. However, seasonal variations may still affect recovered eDNA concentrations in relation for example with growth activity, grazing or decay, as has also been seen for other taxa e.g. fish [15] and amphibians [71]. The time series performed in this study during the months of May through to October clearly shows seasonal variation of detected eDNA for *E*. *canadensis* at a given site. Relative quantities, from the lowest measure obtained in June, increased by more than three log base 10 to reach its maximum during the last sampling month in October. We believe this is explained by the plant’s biomass reaching its peak as well as onset of decaying during fall thereby releasing more DNA into the environment. Similar phenomena may explain the second smaller peak obtained at spring with the first sampling, due to plant survival under ice-covered lakes, in agreement with earlier studies also showing biomass collapse in spring [41, 42]. Differences between surface and bottom sampling at a same date and place were also consistently observed showing higher eDNA values with the bottom samples, including when turbidity limited the possible filtrated volume from 5 L to 0.75 L for the October bottom sample.

## Conclusions

The autumn (October) seems to be the best period for sampling as plant biomass is at its peak with onset of decay, which showed in this study detected eDNA quantities about 1000 times higher than the lowest point observed when sampling during the month of June. Turbidity due to clay particles did not hamper eDNA detection and the rate of disappearance was in the range of one Log_10_ eDNA per km in the stream. These results are paramount for maximizing the efficiency of field surveys planning to map *E*. *canadensis* in possible new locations where quantities may be low, i.e. at the start of a colonization event. It is probable that many aquatic plants may show similar trends, which should always be taken into consideration when planning an eDNA survey.

## Supporting information

S1 Fig Calibration curve. Calibration curve using tenfold serial dilution *E*. *canadensis* genomic DNA starting at 8.6 ng/μL with triplicate technical replicates.
(PDF)

S2 Fig Lake Steinfjorden. The sampling site (red symbol) is shown with the area cover of *Elodea canadensis* in 2004 (Mjelde et al 2012), similar to what was observed in 2017 (Demars, personal observation).
(PDF)

S1 Table Field surveys. Field surveys results for *Elodea canadensis* in the Leira catchment area. Geographic coordinates (EU89) in degrees.
(PDF)

S2 Table Assays from the Hydrocharitaceae family. (PDF)

S1 File Fasta sequences from BLAST Alignments. Alignment of relevant sequences obtained by performing a BLAST search using the *Elodea canadensis* product sequence located on the intergenic spacer between *trn*L and *trn*F. Species specific oligonucleotides are shown at the bottom.
(PDF)

S2 File Assumptions and calculations of decay rates. (PDF)
