# Supplementary material for: Detection of an invasive aquatic plant in natural water bodies using environmental DNA
Source: PLoS One. 2019 Jul 12;14(7):e0219700. doi: 10.1371/journal.pone.0219700 (PMC6625730; doi:10.1371/journal.pone.0219700)

Detection of an invasive aquatic plant in natural water bodies using environmental DNA

Anglès d'Auriac MB, Strand DA, Mjelde M, Demars BOL, & Thaulow J

Supporting information

S1 Fig. Calibration curve. Calibration curve using tenfold serial dilution *E. canadensis* genomic DNA starting at 8.6 ng/µL with triplicate technical replicates.

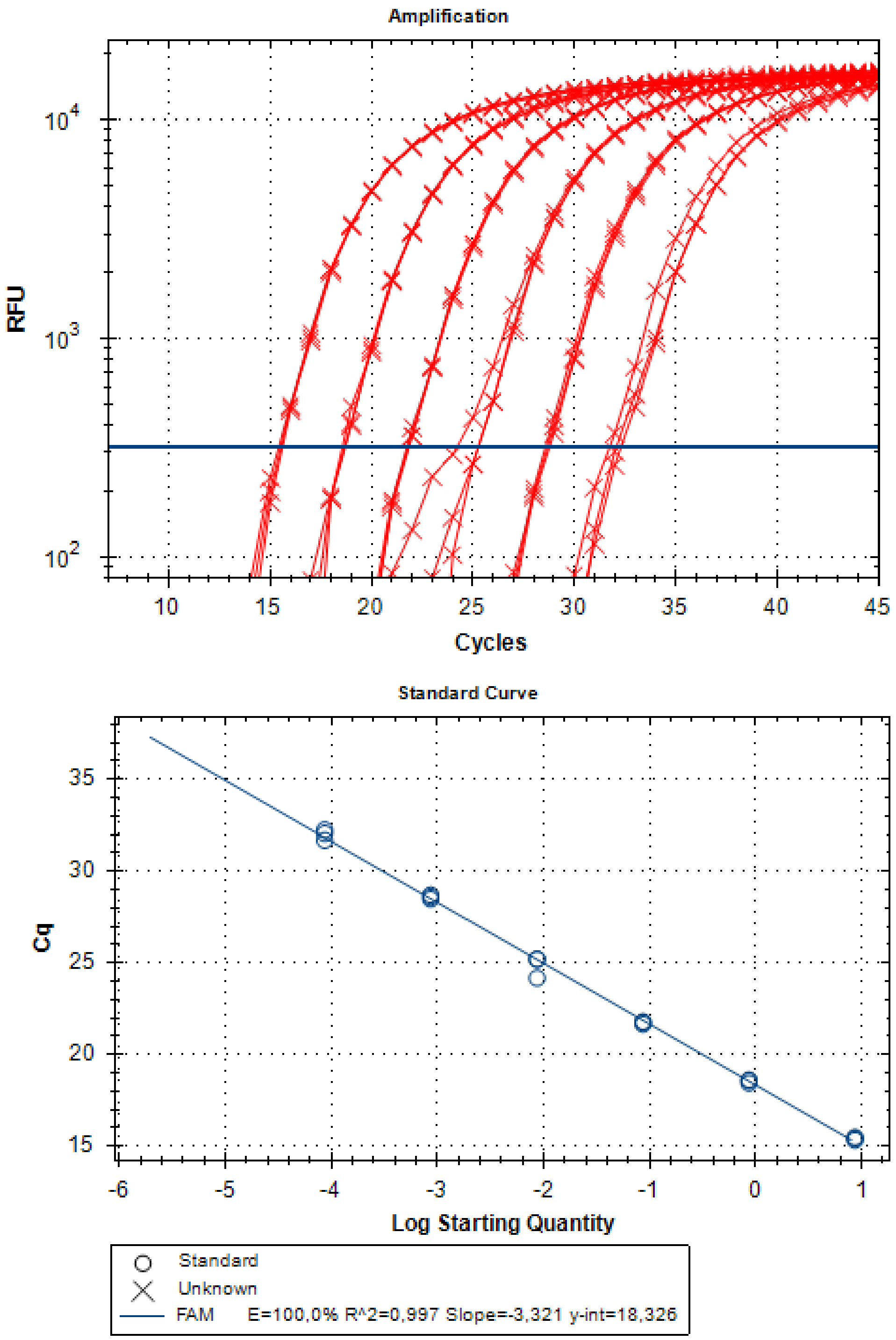

Supplement: S1 Fig — Calibration curve using tenfold serial dilution E. canadensis genomic DNA starting at 8.6 ng/μL with triplicate technical replicates. (PDF) [file pone.0219700.s001.pdf]
