# Supplementary material for: Detection of an invasive aquatic plant in natural water bodies using environmental DNA
Source: PLoS One. 2019 Jul 12;14(7):e0219700. doi: 10.1371/journal.pone.0219700 (PMC6625730; doi:10.1371/journal.pone.0219700)
Supplement: S2 Fig — The sampling site (red symbol) is shown with the area cover of Elodea canadensis in 2004 (Mjelde et al 2012), similar to what was observed in 2017 (Demars, personal observation). (PDF) [file pone.0219700.s002.pdf]

Detection of an invasive aquatic plant in natural water bodies using environmental DNA

Anglès d'Auriac MB, Strand DA, Mjelde M, Demars BOL, & Thaulow J

Supporting information

S2 Fig. Lake Steinfjorden. The sampling site (red symbol) is shown with the area cover of *Elodea canadensis* in 2004 (Mjelde et al 2012), similar to what was observed in 2017 (Demars, personal observation).

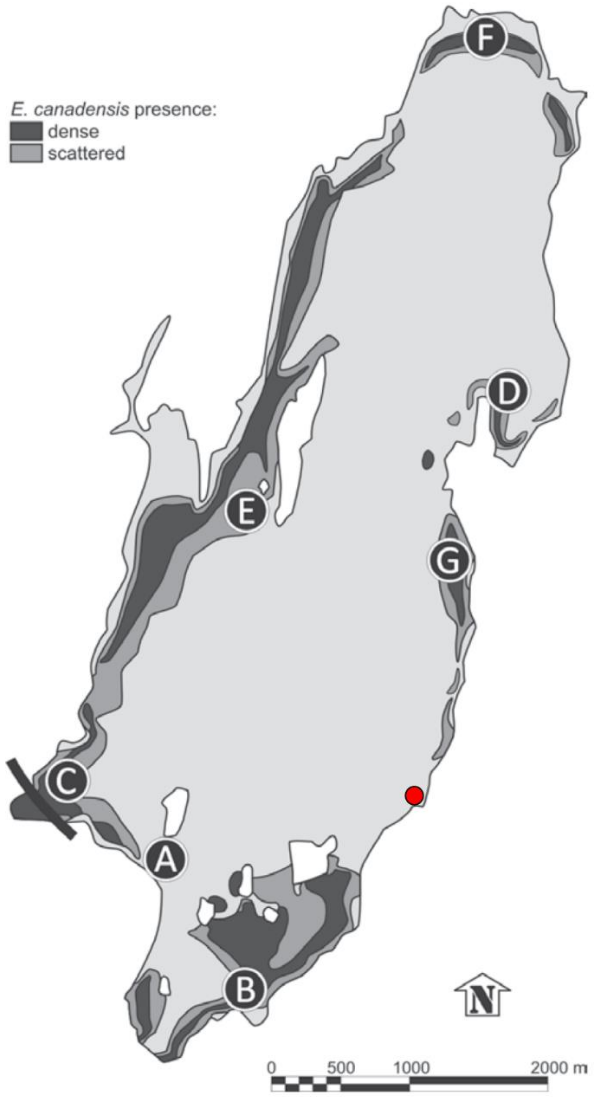

Reference

Mjelde M, Lombardo P, Berge D, Johansen SW. Mass invasion of non-native Elodea canadensis Michx. in a large, clear-water, species-rich Norwegian lake - impact on macrophyte biodiversity. Annales de Limnologie-International Journal of Limnology 2012; 48: 225-240. https://doi.org/10.1051/limn/2012016
