# Supplementary material for: Detection of an invasive aquatic plant in natural water bodies using environmental DNA
Source: PLoS One. 2019 Jul 12;14(7):e0219700. doi: 10.1371/journal.pone.0219700 (PMC6625730; doi:10.1371/journal.pone.0219700)
Supplement: S1 Table — Field surveys results for Elodea canadensis in the Leira catchment area. Geographic coordinates (EU89) in degrees. (PDF) [file pone.0219700.s003.pdf]

Detection of an invasive aquatic plant in natural water bodies using environmental DNA

Anglès d'Auriac MB, Strand DA**,** Mjelde M, Demars BOL, & Thaulow J

Supporting information

S1 Table. Field surveys. Field surveys results for *Elodea canadensis* in the Leira catchment area. Geographic coordinates (EU89) in degrees.

Site	Date	Site name	Occurrence of *Elodea*	Latitude	Longitude
**1**	09.08.2018	Leira (Breenvegen)	absent	60.2535285	11.0047371
**2**	09.08.2018	Leira (Eiksvad bru)	absent	60.1715632	11.0296680
**3**	09.08.2018	Leira (Kråkfoss)	absent	60.1329602	11.0802854
**4**	09.08.2018	Kværndalsbekken	absent	60.1555710	11.1578630
**5**	09.08.2018	Tveia (Gropavegen)	absent	60.1479309	11.1538417
**6**	09.08.2018	Ljøgodttjern naturreservat	absent	60.1477098	11.1383513
**7**	09.08.2018	Tveia (Nordre Haga)	absent	60.1217685	11.1129639
**8**	09.08.2018	Leira (Kløfta)	absent	60.0779336	11.1031764
**9**	09.08.2018	Gjermåa (Svensrud bru)	absent	60.0766315	11.0621212
**10**	09.08.2018	Gjermåa (Tangen)	absent	60.0502266	11.0869730
**11**	09.08.2018	Leira (Eidsvoll)	absent	60.0306541	11.0968809
**12**	09.08.2018	tilførselsbekk v. Øvre Myrer	absent	60.0081973	11.1127424
**13**	09.08.2018	Leira (Leirsund)	absent	59.9975372	11.0885309
**14**	09.08.2018	Tomtestilla	absent	59.9836284	11.0788106
**15**	10.08.2018	Bakevje v. Tuen	absent	59.9391977	11.0882452
**16**	10.08.2018	Jølsenbekken	absent	59.9544312	11.0997203
**17**	10.08.2018	Mastevika	absent	59.9441419	11.0875198
**18**	10.08.2018	Andevika	absent	59.9426844	11.0827755
**19**	01.09.2018	Stilla	absent	59.9629710	11.0758797
**20**	01.09.2018	Ringstilla	absent	59.9703652	11.0840734
**21**	09.09.2018	Isakbekken	present	59.9481599	11.0834373
**22**	09.09.2018	Lille Sandhølet	absent	59.9479357	11.0861835
**23**	14.10.2018	Dam v Jessheim (skibakke)	absent	60.1510217	11.1591831
