# Supplementary material for: Detection of an invasive aquatic plant in natural water bodies using environmental DNA
Source: PLoS One. 2019 Jul 12;14(7):e0219700. doi: 10.1371/journal.pone.0219700 (PMC6625730; doi:10.1371/journal.pone.0219700)
Supplement: S2 Table — (PDF) [file pone.0219700.s004.pdf]

Detection of an invasive aquatic plant in natural water bodies using environmental DNA

Anglès d'Auriac MB, Strand DA, Mjelde M, Demars BOL, & Thaulow J

Supporting information

S2 Table. Assays from the Hydrocharitaceae family.

**Species**	**Native-range**	**Known invaded-range**	**Locus**	**Reference**
Elodea canadensis Michx.	North America	Europe	*trn*L–*trn*F	This study
			ITS1	[1]
			*mat*K*	[1]
Elodea nuttallii (Planch.) St-John	North America	Europe	ITS1	[1]
			*mat*K*	[1]
Egeria densa Planch.	South America	North America, Europe, Asia, Australia, New Zealand and Africa	*mat*K	[2]
			*trn*L–*trn*F	[3]
Hydrilla verticillata (L.f.) Royle	Asia, Africa and Australia	North America	ITS1	[1]
			*mat*K	[1, 4]
Lagarosiphon major (Ridley) Moss	South Africa	New Zealand, Europe	-	-

*Same assay which indistinctively amplifies *E. canadensis* and *E. nuttallii*

References

1. Gantz CA, Renshaw MA, Erickson D, Lodge DM, Egan SP. Environmental DNA detection of aquatic invasive plants in lab mesocosm and natural field conditions. Biol Invasions. 2018; 20(9):2535-52. doi: 10.1007/s10530-018-1718-z. PubMed PMID: WOS:000441112500018.
2. Scriver M, Marinich A, Wilson C, Freeland J. Development of species-specific environmental DNA (eDNA) markers for invasive aquatic plants. Aquat Bot. 2015; 122:27-31. doi: 10.1016/j.aquabot.2015.01.003. PubMed PMID: WOS:000352172400005.
3. Fujiwara A, Matsuhashi S, Doi H, Yamamoto S, Minamoto T. Use of environmental DNA to survey the distribution of an invasive submerged plant in ponds. Freshw Sci. 2016; 35(2):748-54. doi: 10.1086/685882. PubMed PMID: WOS:000376471600025.
4. Matsuhashi S, Doi H, Fujiwara A, Watanabe S, Minamoto T. Evaluation of the environmental DNA method for estimating distribution and biomass of submerged aquatic plants. PLoS One. 2016; 11(6):e0156217. doi: 10.1371/journal.pone.0156217. PubMed PMID: WOS:000377824800014.
