# Supplementary material for: Detection of an invasive aquatic plant in natural water bodies using environmental DNA
Source: PLoS One. 2019 Jul 12;14(7):e0219700. doi: 10.1371/journal.pone.0219700 (PMC6625730; doi:10.1371/journal.pone.0219700)
Supplement: S1 File — Alignment of relevant sequences obtained by performing a BLAST search using the Elodea canadensis product sequence located on the intergenic spacer between trnL and trnF. Species specific oligonucleotides are shown at the bottom. (PDF) [file pone.0219700.s005.pdf]

Detection of an invasive aquatic plant in natural water bodies using environmental DNA

Anglès d'Auriac MB, Strand DA, Mjelde M, Demars BOL, & Thaulow J

Supporting information

S1 File. Fasta sequences from BLAST Alignments. Alignment of relevant sequences obtained by performing a BLAST search using the *Elodea canadensis* product sequence located on the intergenic spacer between *trn*L and *trn*F. Species specific oligonucleotides are shown at the bottom.

>KP263412_Hydrilla-verticillata; Hydrilla verticillata isolate HvrPPR2 tRNA-Leu (trnL) gene, partial sequence; trnL-trnF intergenic spacer, complete sequence; and tRNA-Phe (trnF) gene, partial sequence; chloroplast

TTTCTCGTTCACTCTACTCTATTTACAAACGACGGATCCGAACAGAAATGCTCATATCTT

ATCCCACCTCAAGTTTTTGCTGGCTACAATACACATAGAAATCCATA

>KP676540_Ottelia-emersa; Ottelia emersa isolate H18 trnL-trnF intergenic spacer region and tRNA-Phe (trnF) gene, partial sequence; chloroplast

TTTCTCGTTCATTCTACTCT-TTCACAAGCG---GGTCCGAACAGAAATGCTTCTATCTT

ATCCCATCCTAAGTTTTTGGTG--------GCACATAGAAATCAACA

>KP676525_Ottelia-acuminata; Ottelia acuminata isolate H10 trnL-trnF intergenic spacer region and tRNA-Phe (trnF) gene, partial sequence; chloroplast

TTTCTCGTTCATTCTACTCT-TTCACAAGCG---GGTCCGAACAGAAATGCTTCTATCTT

ATCCCATCTTAAGTTTTTGGTG--------GCACATAGAAATCAACA

>KY554851_Ottelia-balansae; Ottelia balansae isolate H22 trnL-trnF intergenic spacer and tRNA-Phe (trnF) gene, partial sequence; chloroplast

TTTCTCGTTCATTCTACTCT-TTCACAAGCG---GGTCCGAACAGAAATGCTTCTATCTT

ATCCCATCTTAAGTTTTTGGTG--------GCACATAGAAATCAACA

>JF703282_Elodea-nuttallii; Elodea nuttallii isolate SAV93 trnL-trnF intergenic spacer, partial sequence; chloroplast

TTTCTCCTTCATTGTATTCT-TTCACAAGCG---GGTCCGAACAGAAATGC--------T

ATCCCATCCCAAGGTTTTGG---------TACAGATAGAAATCAACA

>JF703276_Egeria-densa; Egeria densa isolate SAV63 trnL-trnF intergenic spacer and tRNA-Phe (trnF) gene, partial sequence; chloroplast

TTTCTCCTTCATTGTATTCT-TTCACAAGCG---GGTCCGAACAGAAATGCTTCTCTCTT

ATCCCATCCCAAGGTTTTGGTGGCTACGATACAGATAGAAATCAACA

>JF703283_Elodea-canadensis; Elodea canadensis isolate SAV94 trnL-trnF intergenic spacer, partial sequence; chloroplast

TTTCTCCTTCATTGTATTCT-TTCACAAGCG---GGTCCGAACAGAAATGCCTCTCTCTT

ATCCCATCCCAAGGTTTTGGTGTCTACAATACAGATAGAAATCAACA

>EctrnL_Primers-&-Probe 26 Weight: 1.24

TTTCTCCTTCATTGTATTCT-TTCACA---------TCCGAACAGAAATGCCTCTCTCTT

ATCC-----------------GTCTACAATACAGATAGAAATCAACA
