# Supplementary material for: Detection of an invasive aquatic plant in natural water bodies using environmental DNA
Source: PLoS One. 2019 Jul 12;14(7):e0219700. doi: 10.1371/journal.pone.0219700 (PMC6625730; doi:10.1371/journal.pone.0219700)
Supplement: S2 File — (PDF) [file pone.0219700.s006.pdf]

Detection of an invasive aquatic plant in natural water bodies using environmental DNA

Anglès d'Auriac MB, Strand DA**,** Mjelde M, Demars BOL, & Thaulow J

Supporting information

S2 File. Assumptions and calculations of decay rates.

If we assume a steady state flow of eDNA and an exponential rate of decay [1], then *eDNA* = *b* exp(-*rL*), with *eDNA* (fg mL^{-1}), *L* distance (m) from lake outlet, *b* initial concentration (fg mL^{-1}) and *r* the rate of disappearance (m^{-1}). The reciprocal of the rate of disappearance S_{net}=1/*r* gives the average distance (m) over which a detectable fragment of eDNA travels before it is lost either by adsorption on the benthos, degradation or dilution beyond the detection limit. In the stream S_{net} is the net uptake length or spiralling length [2].

1. Shogren AJ, Tank JL, Egan SP, August O, Rosi EJ, Hanrahan BR, et al. Water Flow and Biofilm Cover Influence Environmental DNA Detection in Recirculating Streams. Environmental Science & Technology. 2018; 52(15):8530-7. doi: 10.1021/acs.est.8b01822. PubMed PMID: WOS:000441477600052.
2. Haggard BE, Storm DE, Stanley EH. Effect of a point source input on stream nutrient retention. JAWRA Journal of the American Water Resources Association. 2001; 37(5):1291-9. doi: doi:10.1111/j.1752-1688.2001.tb03639.x.
